# A Clinical Lecture Delivered at the Hospital of the Sisters of Charity, Jan. 13th, 1883

**Published:** 1883-02

**Authors:** W. S. Tremaine

**Affiliations:** U. S. A.


### Clinical Reports.

### A Clinical Lecture Delivered at the Hospital of the Sisters of Charity, Jan. 13th, 1883.

By W. S. Tremaine, M. D., U. S. A.

Gentlemen—The first patient I show you to-day is the man you may recollect having seen before and who is suffering with cystitis. I think it desirable in clinics that students should be shown the result of treatment. It is quite the fashion to operate, or to describe a case, and order some treatment, and then the class never sees it again and knows nothing of the results. I think the effects of the treatment should be shown, whether it be good or bad, and the progress of the case followed, because that is the proper way to obtain clinical knowledge of disease.

This man, whom we found had cystitis and whose urine was highly alkaline, has somewhat improved. When we first saw him, he was obliged to pass his urine about 24 times during the night. He now says that he only has to pass it about six times, and passes it in a larger quantity. He tells me that his original trouble has improved. Here is some of his urine. You see that it still contains some sediment, but not as much as before. I told you that in cystitis the urine was alkaline, on account of ammoniacal decomposition going on. We find that it is now acid, and in so far it has improved. The patient now complains of pain in the lumbar region, and that he was doing well, but within the last few days he has had what he called a "set back." I take it, from the region to which this man refers his pain, that there possibly is some pyelitis, that is, inflammation of the pelvis of the kidney. I yesterday examined some of this urine, microscopically, and found it loaded with pus cells. You cannot rely entirely upon the results obtained by microscopical examinations, but, as your distinguished professor of obstetrics told you yesterday morning, the microscope is a great aid in diagnosis.

I take it that there is some pyelitis, which may have come from the original trouble from which the man was suffering, but I have not time, to-day, to dwell upon this case or to repeat the points I have already given you upon this subject.

I merely show you this man to follow out the idea which I believe to be the true one, that students should be shown the results of the treatment ordered in cases brought before them. You see there is some improvement. The only object in showing it is to demonstrate that under the treatment of washing out the bladder the alkaline decomposition can be prevented. There is every reason to believe, from the improvement which has taken place, that the man will get well eventually.

I believe that clinical lectures should be confined to a few cases, and the symptomatology and the diagnosis should be amplified as much as possible, taking the cases as the text. In that way a few cases can be made more useful than a greater number in which you merely witness surgical operations.

I find in the ward a case which I present to you to-day for two reasons. In the first place, there is a condition of affairs which you will frequently meet with. Secondly, it contains instructive lessons in diagnosis.

As I have had occasion to say before, diagnosis is the most important art that you can acquire. It is an art to be acquired by long and patient observation of the phenomena of disease. What is it? It is the examination of the evidence and a legitimate verdict drawn therefrom.

That quality of mind which makes a man a good diagnostician, will make him a good judge. In the case of a patient we have the evidence obtained from the physical characteristics, and we have also the oral testimony given by the patient, or sometimes by friends. This oral testimony must be corrected by the physical examination and by the knowledge which the surgeon possesses of the function of the parts and the diseases to which they are liable. I say this because patients will lie to you, consciously and unconsciously. I lay stress upon the importance of the art of diagnosis, because it is only in a clinic that you can learn it. Hereafter you will learn from examination of your own patients, relying on your own intelligence; but intelligence is of very little value, unless you have also intelligent experience. To learn to be good diagnosticians, then, is of great importance to you. You can learn a great deal in regard to treatment from your text-books, but not in regard to diagnosis. You must be told and shown what to observe and where to look for the lesions. It is not only necessary to become good diagnosticians, but necessary, also, to make a rapid diagnosis. You cannot leave a case and run to your books to consult authorities, so that you must be able to make a rapid and accurate diagnosis. I say accurate, approximately, because you can only approach more and more nearly to accuracy as your years of experience increase. You will never attain *perfection*, but you must constantly strive after it. This is why the opinion of age and experience, other things being equal, is of so much value, especially in surgery.

Before investigating that portion of the body of which the patient complains, it is, perhaps, well to refresh your memories regarding the anatomy and functions of that part. When we observe this patient we see there is a deviation from the normal in the vicinity of the knee-joint. I say in the vicinity of the knee-joint, because we have not determined whether it is inside of the joint or not. Let me first recall to your recollection some of the anatomical features of this region. First, we have the skin; beneath that the superficial fascia, and in this place a bursa; then the patella, and again a closed sac, called the synovial membrane, which has a special function. Then we have the cartilages and the ligaments; or, first of all, the ligaments, white fibrous tissue and very inelastic. In addition to that we have fibro-cartilages, then the ends of the bones composing the knee-joint. This joint has the largest synovial membrane of any in the body. Upon inspection, we find a swelling in this neighborhood that may be confined to any one or may embrace all of these structures. First, in regard to the skin, when I pinch up the skin I find it compares very favorably with the other side, except that there is some removal of the epidermis, which is probably due partially to pressure and partially to counter-irritation. Then I find not much the matter with the skin, so that does not account for the injury or loss of function that this man complains of in this region. Suppose that this swelling was confined to the skin or subcutaneous or areolar tissue, the next step would be to ascertain the nature of it. Knowing, as we should know, the function of these parts, we are aware that serous effusion in the cellular tissue very frequently takes place, and is called œdema. I find that there is no pitting upon pressure, nor when I press upon it can I disperse the fluid, as I could do were this fluid in the areolar tissue. I ascertain, first, that it is probably fluid that causes this swelling; when I go further I find that it gives a sensation of fluctuation. In addition to that, when I press down upon the patella I get what is called knocking of the patella. That is, when I press down upon it and bring it in contact with the condyles of the femur, I get a characteristic feeling, which is called knocking, which I am not able to produce in the patella of the sound leg. The patella is lifted up as it was, then recalling your anatomy and knowing that the patella enters into the formation of the knee-joint and is covered with synovial membrane, I think it is probable that there is fluid in the joint.

Before proceeding further it is well to ascertain by measurement the difference in the two limbs. Always compare the diseased limb with the sound one. Measuring, then, at the knee, I find that the diseased one is an inch and a half larger in circumference than the sound one. Again, when I measure at the thigh, above the knee, I find that the sound limb is two inches larger than the other. Why is this? I think the condition of the knee-joint throws some light upon it. We have ascertained that there is a probability of fluid in the cavity of the synovial membrane; that is, in the cavity of the knee-joint, lined by synovial membrane. How did that get there? What is the cause of it? Let us go further and get something of the history of the case. I will give it to you as the patient repeats it to me. Upon the 7th of November he received a severe injury, in this way: He was on board a vessel on the lake, and what is called the main-sheet, which, by the way, is not a sheet but a rope, caught him by the leg and jammed him against a block. As a result of that, he had severe pain and enormous swelling in this region. In such an injury it is well to inquire into the cause, in order to estimate the amount of force, and thus, to some extent, the amount of injury to the tissues. Had the history been that a car-wheel had passed over there I should have expected to find a different condition of affairs. Had that limb been crushed between two buffers of a car I should also have expected to find a different condition; so I allude to the history.

You will understand from what I have said regarding the way the injury was produced that there was a great deal of violence. The result was that a large swelling took place, much larger than at present. He further tells me that for ten days after that he had no treatment, and then he saw Dr. Heath, who treated this limb and brought it down to the present size, or rather, aided Nature to do so. Such is the history of the case, and I have indicated what the probable condition is. Now, what is that? Naturally you will say some disturbance in the synovial membrane, and being the result of violence you will probably say it is inflammation of the synovial membrane, and that it has resulted in increased effusion and alteration in character of the synovial fluid. But I spoke to you in regard to the difference in size of these limbs. You notice that the muscles of this limb are much smaller than those of the sound limb. Is there anything to account for this? It is noticeable, in this disease, that wasting of the muscles takes place rapidly, but in my judgment this difference is not entirely accounted for by the injury to the knee-joint. You see there is a flattening of the gluteal muscles. The leg that is diseased is shorter than the other. Now the history of this case is that the man received an injury in childhood, and when he grew older his parents took him to a surgeon, and the surgeon stated that a fracture had taken place in the neck of the femur, and that it was too late to remedy it. I think that is probably the case. There is no history of hip-joint disease, and probably it is this old accident that has rendered this limb smaller than the other. So you must go farther than appears on the surface in all these matters, knowing, as you should know from your studies, that atrophy of the limb does take place in injuries of the hip, especially when occurring in childhood. In measuring the two limbs to compare them, remember that you will have to get the pelvis at right-angles to the axis of the body. Approximately, then, when we measure these limbs from the umbilicus to the border of the internal malleolus, we find a difference of an inch and three-quarters. We also find talipes varus. What caused that? That is due to the fact that the atrophy of the muscles has taken place somewhat irregularly, and that the action of the tibialis anticus has overcome the action of the peronei muscles, and that is the way in which talipes varus takes place. But to return to the knee-joint. In examining further you notice that on each side of the sound patella there is a furrow or pit, which, in the diseased limb, is obliterated. The synovial membrane is not a tight sac, but has certain folds and pouches, the ligamentum mucosum, and on each side of the patella the ligamenta alaria, not ligaments, but reflections of synovial membrane, a process or pouch extends up beneath the extensor tendon, and into which, you will remember, the subcranus muscle is inserted; its use is to pull this pouch out of the way when the leg is extended.

You will readily understand that when the synovial sac has an increased amount of fluid in it, that these folds are more or less unfolded, and the pouches distended; in this case you see the result; the inequalities of the joint are obscured and a rounded, globular outline given. Now observe, when I place the palm of one of my hands lightly on one side of the joint and tap gently with the other hand on the opposite side, I feel a peculiar impulse from a wave of fluid; this sensation is very characteristic, when you are once familiar with it. I feel now quite satisfied that there is fluid in the joint, and taking into consideration the history of the case, and all the symptoms, our diagnosis is complete—synovitis; in its earlier stages, "simple acute synovitis," now chronic.

A few words with regard to the nature of this synovial inflammation. Many causes contribute to it; over-exertion, strains, violence (as in this case), the rheumatic and strumous diathesis, syphilis, absorption of pus, a form of which results occasionally from gonorrhœa.

The nomenclature depends somewhat upon the cause, and sometimes from the character of the fluid; thus we have traumatic and rheumatic synovitis; again, according to the nature of the fluid, "simple synovitis" and "purulent."

Let us glance for a moment at the pathology. When an irritation, from any cause sufficient to excite inflammation, takes place, the first effect is, of course, hyperemia and increased secretion of the synovial fluid; later, the engorged vessels relieve themselves by serous effusion, the normal endothelial cells are thrown off, white and red blood corpuscles wander out of the vessels, sometimes fibrin is formed. If the fluid be withdrawn and examined, it shows a milky or opalescent appearance; the degree of this, of course, varies as the cellular or serous element predominates.

The villous-like fringes enlarge; this takes place in a more marked degree in a form of the disease of which we have not time to go into to-day. When fibrin is more abundant, it becomes partially organized, adherent to the synovial membranes, constituting what is sometimes called "synovitis sicca," or dry synovitis. But a history of these changes would involve the whole pathology of inflammation, which you must study elsewhere. The subject is too interesting and too vast to include in one clinical lecture. Study it when you have time. You will be amply repaid.

I forgot to show you, before the patient left, the amphitheatre; that pain could be produced by extension of the limb. This is not clearly explained. Perhaps you know that we do not often have an opportunity to investigate these cases pathologically, as death of the patient does not often result; I am speaking of simple forms of the disease, but it is believed that this condition is brought about because there is a little lump of fibrin or portion of the inflamed synovial membrane that is pinched. There is also another reason which has occurred to me. You know there is a pouch there. Now, you understand that when that is in a normal condition, no pain will take place, but when it is inflamed and there is pressure upon it, pain will be produced.

Let us make a rough outline of the symptoms of synovitis. Swelling you always find. Redness, one of the other symptoms of inflammation, not always accompanies it. In fact, in acute simple synovitis it is not generally present. In the rheumatic form it is very characteristic. In the pyæmic form redness of a dark bluish cast is very common. In some of the strumous varieties the redness is something which is unmistakable in these cases. Now, with regard to pain: Always present, and generally of a very acute kind. Perhaps nothing gives more pain than a simple acute attack of synovitis. Immobility is another characteristic. There is, too, a peculiarity about this; there is a tendency to contraction of the flexor muscles, simply because the extensor muscles are more easily tired out than the flexors.

Another symptom which I pointed out to you was the wasting or atrophy of the muscles which takes place. So far as my reading goes there is no very pronounced explanation given of this. In some way which we do not clearly understand atrophy takes place: It is possibly due to the fact that the muscles of the thigh derive their innervation from the same source as the structures of the joint, and by the irritation of the terminal filaments, trophic changes are produced; but you always find it takes place, and the longer the condition lasts the more marked will be the atrophy of the muscles.

Now, I have spoken of one form, simple acute synovitis. This may terminate in recovery; the fluid becomes absorbed; the cells undergo fatty degeneration, are found filled with fatty granules; these undergo absorption and the disease disappears. But, does the joint always resume its normal function? I think not. There is often a puffiness or distension, which leaves the joint in a relaxed and weakened condition. One result that these patients often complain of is that in making a sudden motion severe pain is induced. This relaxed state leaves the joint in a weakened condition, and they frequently seek treatment for it.

There are other forms to which I will ask your attention for a moment. You will readily understand that when inflammation of the synovial membrane occurs it may terminate, under ordinary good treatment, in a cure, or it may go on to suppuration; but suppose we have associated with it a peculiar diathesis, and we will allude more particularly to that called the "strumous." This exudation remains and becomes more milky and filled with flocculi, and the destruction of the tissue goes on, the separation of the membrane from the cartilages beneath, and finally erosion of the cartilages, and destruction, more or less, of the functions of the joint. This was formerly known by the name of "White Swelling," a very indefinite term. In this strumous synovitis, when examined pathologically, we find the gelatiniform exudation, destructive changes go on with necrosis of cartilages, osteitis of the articular ends of the bones, and possibly the case may terminate even in death of the body.

Now, with regard to treatment. My own judgment is that the sooner the fluid is evacuated the better. For that reason I commend to you the early evacuation of the contained fluid, but with certain precautions. You know that if you admit air into the joint, ordinarily, you will have resulting suppuration.

In former times this opening was made by valvular incision, by a long narrow knife; the synovial membrane was opened at the inner border of the patella and the fluid dispersed through the subcutaneous tissue. But since the invention of the aspirator we have a better method. Before using the aspirator, dip it into carbolic acid, and see that in its introduction no air is allowed to enter the joint. It is objected to this that the fluid recurs. If it does, aspirate again, and again; "tire it out." If it does it certainly would not get well under ordinary treatment. But you may go still further, you may stimulate or alter the nutrition of this membrane by injection of fluids which produce irritation; but do this with great care. You may inject a few drops of diluted iodine, and then by care prevent excessive resulting inflammation. The advantage of compression in producing absorption of fluids is very great. So, another method is by putting on pressure. One very good way is by compressed sponges. Another way is by the application of the rubber bandage, a solid rubber bandage. In the acute form of the disease one of the most important matters is to keep the joint in perfect rest. On the continent of Europe, they are in favor of the plaster of Paris bandage. I dislike that, because as the swelling goes down the plaster does not follow it and keep up the pressure. I also prefer that the joint should be open for inspection from day to day. I prefer that it should be slightly flexed on a double-inclined plane. The object of this is, that if anchylosis takes place the limb will be in better shape. It is also more comfortable for the patient. You cannot, by putting it on a pillow, insure rest. The administration of morphine will be necessary, of course, to quiet pain. Another form of splint is Hodgen's (this splint and its application was described), which is a modification of "Smith's anterior." When this is applied the whole apparatus is suspended by a cord to the ceiling, and the limb immobilized in that way. This gives great relief and enables the patient to move about considerably. I am in the habit of using that form of splint, and consider it decidedly preferable to the plaster of Paris apparatus. If you prefer to use plaster, the best way is to use a posterior splint formed of several thicknesses of blankets or coarse flannels. To facilitate its setting rapidly, add alum to the plaster. (The manner of making this splint was shown.)

So much for the ordinary acute synovitis. When it becomes chronic, as in this case, I think puncture, that is, aspiration, is the best mode of procedure. Whether the fluid will be completely absorbed under the present treatment, I think is doubtful. There are other methods, stimulating plasters, mercurial plasters, etc. I think, in regard to liniments, that the chief benefit is in the massage, or rubbing; perhaps another advantage is the psychological influences upon the patient, but, I confess, I put very little faith in them.

I will, in a few words, allude to the treatment of the strumous form. When these destructive changes take place, it becomes a question whether the joint should be removed or the limb amputated, and this requires a good deal of judgment on your part. Let me urge upon you to decide the question in time, before your patient is exhausted. I recall a case where the man was very averse to having his limb amputated, and after months of suffering, the poor fellow was worn out and died. So that when these destructive changes go on, and erosion of the cartilages takes place, it becomes a question whether the joint shall be exsected or the limb amputated. I urge upon you, when you find this condition of things, to do something, otherwise the patient is likely to be worn out. Some operative surgery is necessary. The form of synovitis which produces these changes, occurs, as a rule, only in strumous subjects. This has been disputed. It has been stated that strumous synovitis never occurs, except as the result of an injury. I think it can occur in a certain class of cases which have a certain diathesis which we call the strumous. I do not go into the question of opening the joint under antiseptic precautions and removing the diseased structure; this I have done with success, and it is now quite frequently practiced; but the hour has expired and I must dismiss you.

